# Correction: Messele et al. Longitudinal Analysis of Antimicrobial Resistance among *Enterococcus* Species Isolated from Australian Beef Cattle Faeces at Feedlot Entry and Exit. *Animals* 2022, *12*, 2690

**DOI:** 10.3390/ani13162684

**Published:** 2023-08-21

**Authors:** Yohannes E. Messele, Mauida F. Hasoon, Darren J. Trott, Tania Veltman, Joe P. McMeniman, Stephen P. Kidd, Wai Y. Low, Kiro R. Petrovski

**Affiliations:** 1 The Davies Livestock Research Centre, School of Animal and Veterinary Sciences, University of Adelaide, Adelaide, SA 5005, Australia; 2 The Australian Centre for Antimicrobial Resistance Ecology, University of Adelaide, Adelaide, SA 5005, Australia; 3 Meat & Livestock Australia, Level 1, 40 Mount Street, North Sydney, NSW 2060, Australia; 4 Research Centre for Infectious Disease, School of Biological Sciences, University of Adelaide, Adelaide, SA 5005, Australia

## Text Correction

There was an error in the original publication [1]. In the simple summary conclusion, “…to combat the antimicrobial-resistant infections” should be replaced with “…to monitor resistance trends in beef cattle”.

## Table Correction

In Table 1, a correction is needed for the tetracycline resistance breakpoints: “6” should be replaced with “16”.

The authors apologize for any inconvenience this change may cause. The authors state that the scientific conclusions are unaffected because the correction involves a typing error that was not caught during proofreading.

This correction was approved by the Academic Editor. The original publication has also been updated.
