# Supplementary material for: Longitudinal Analysis of Antimicrobial Resistance among Enterococcus Species Isolated from Australian Beef Cattle Faeces at Feedlot Entry and Exit
Source: Animals (Basel). 2022 Oct 6;12(19):2690. doi: 10.3390/ani12192690 (PMC9559632; doi:10.3390/ani12192690)
Supplement: Supplementary file 1 [file animals-12-02690-s001.zip › animals-1944898-supplementary.pdf]

Table S1. Antimicrobial resistance profile of *Enterococcus faecium* and *E. hirae* isolated at entry and exit from the feedlot

**Antimicrobial**	**Sample source and no. (%) of *Enterococcus* isolates**			
	**Feedlot (Entry)**		**Slaughter house (Exit)**	
	***E. faecium* (n=9)**	***E. hirae* (n=90)**	***E. faecium* (n=117)**	***E. hirae* (n=25)**
Chloramphenicol	0	0	0	0
Ciprofloxacin	77.8	0	9.4	0
Daptomycin	11.1	27.8	17.9	48
Erythromycin	11.1	0	2.56	8
Gentamycin	0	0	0	0
Kanamycin	0	0	0.9	0
Lincomycin	33.3	61.1	82.9	84
Linezolid	0	0	0	0
Nitrofurantoin	22.2	7.8	61.5	16
Penicillin	0	0	0	0
Quinupristin/Dalfopristin	22.2	0	21.4	0
Streptomycin	0	0	0.9	0
Tetracycline	33	1.1	5.1	12
Tigecycline	0	4.4	0.9	4
Tylosin tartrate	11	0	1.7	8
Vancomycin	0	0	0	0

Table S2. Antimicrobial resistance pattern in *Enterococcus faecium* and *E. hirae* isolated at entry or exit from the feedlot

Antimicrobial classes	Entry Resistance pattern (%)[a]		Exit Resistance pattern (%)[b]	
	E. faecium (n=9)	*E. hirae* (n=90)	*E. faecium* (n=117)	*E. hirae* (n=25)
All susceptible	0	26 (28.9)	0	4 (16)
1	CIP, 3(33.3)	LIN, 35 (38.9)	LIN, 32 (27.4)	LIN, 6 (24)
	TET, 1 (11.1)	DAP, 6 (6.7)	NIT, 6 (5.1)	
		TIG, 2 (2.2)		
2	CIP-NIT, 1 (11.1)	DAP-LIN 11 (12.2)	LIN-NIT , 26 (22.2)	DAP-LIN, 7 (28)
	CIP-TET, 1 (11.1)	LIN-TIG, 2 (2.2)	LIN-Q/D, 9 (7.7)	ERY-LIN-TYL, 1 (4)
		DAP-NIT, 1 (1.1)	CIP-NIT, 1 (0.8)	LIN-NIT, 1 (4)
			CIP-LIN, 1 (0.8)	
			DAP-NIT, 7(6.0)	
			LIN-TET, 1 (0.8)	
3	CIP-LIN-NIT, 1 (11.1)	DAP-LIN-NIT, 6 (6.7)	DAP-LIN-NIT, 6 (5.1)	DAP-LIN-NIT, 2 (8)
		DAP-LIN-TET, 1 (1.1)	CIP- LIN- NIT, 3 (2.6)	DAP-LIN-TET, 2 (8)
			LIN-NIT-Q/D, 10 (8.5)	ERY-LIN-TIG-TYL, 1 (4)
			DAP-LIN-Q/D, 1 (0.8)	
			NIT-STR-TET, 1 (0.8)	
			CIP-DAP-NIT, 3 (2.6)	
			CIP-NIT-TIG, 1 (0.8)	
			LIN-NIT-TET, 1 (0.8)	
4	ERY-LIN-Q/D-TET-TYL, 1 (11.1)		CIP-DAP-LIN-NIT, 1 (0.8)	DAP-LIN-NIT-TET, 1 (4)
	CIP-DAP-LIN-Q/D, 1 (11.1)		DAP-LIN-NIT-Q/D, 1 (0.8)	
			DAP-ERY-LIN-NIT, 1 (0.8)	
			KAN-LIN-NIT-Q/D, 1 (0.8)	
			LIN-NIT-Q/D-TET, 1 (0.8)	
			ERY-LIN-Q/D-TET-TYL, 1 (0.8)	
			ERY-LIN-NIT-TET-TYL, 1 (0.8)	
5			CIP-DAP-LIN-NIT-Q/D, 1 (0.8)	

MDR (%)	3(33.3)	7 (7.8)	34 (29.1)	6 (24)
Resistance (%)	9(100)	64(71.1)	117(100)	21 (84)

[a] Isolates from entry; [b] Isolates from exit; CIP, Ciprofloxacin; DAP, Daptomycin; ERY, Erythromycin; KAN, Kanamycin; LIN, Lincomycin; NIT, Nitrofurantoin; Q/D, Quinupristin/dalfopristin; STR, Streptomycin; TET, Tetracycline; TIG, Tigecycline; TYL, Tylosine tartrate

Table S3. The antimicrobial resistance phenotype and genotype of 62 *E. faecium* isolates obtained at entry and exit from feedlot cattle faecal samples and subjected to whole genome sequencing analysis

Antimicrobial classes pattern	Total no. of isolates (%)		Resistance pattern (no. of isolates)	
	**Phenotypic**	**Genotypic**	**Phenotypic (MIC)**	**Genotypic(resistance gene)**
1	20 (32.2)	2 (3.2)	CIP (3)<br>DAP (13)<br>LIN (4)	*aac(6′)-Iid* (2)
2	13(21.0)	0	CIP-LIN (1)<br>CIP-NIT (2)<br>CIP-TET (1)<br>Q/D-LIN (9)	
3	19 (30.6)	28 (45.2)	CIP-LIN-NIT (4)<br>CIP-DAP-NIT (3)<br>CIP-TIG-NIT (1)<br>DAP-Q/D-LIN (1)<br>Q/D-LIN-NIT (10)	*aac(6′)-Ii ,eatAv, msr(C) (25)*<br>*aac(6′)-Ii, pbp5,msr(C)* (1)<br>*aac(6′)-Ii, efmA, pbp5* (1)<br>*eatAv, efmA, msr(C)* (1)
4	9 (14.5)	23 (37.1)	CIP-DAP-LIN-NIT (1)<br>CIP-DAP-Q/D-LIN (1)<br>DAP-ERY-LIN-NIT (1)<br>DAP-Q/D-LIN-NIT (1)<br>Q/D-KAN-LIN-NIT (1)<br>Q/D-LIN-NIT-TET (1)<br>ERY-Q/D-TYL-LIN-TET (2)<br>ERY-TYL-LIN-NIT-TET (1)	*aac(6′)-Ii , eatAv, pbp5, msr(C),* (9)<br>*aac(6′)-Ii , efmA, pbp5, msr(C)* (11)<br>*aac(6′)-Ii , eatAv, lnu(G), msr(C)* (2)<br>*aac(6′)-Ii , eatAv, msr(C), tet(S) (1)*
5	1 (1.6)	5 (8.1)	CIP-DAP-Q/D-LIN-NIT (1)	*aac(6′)-Ii , eatAv, efmA, pbp5, msr(C)* (5)
6		4 (6.4)		*aac(6′)-Ii , eatAv, efmA, pbp5, msr(C), tet(M), (1)*<br>*aac(6′)-Ii , eatAv, efmA, erm(B), msr(C), tet(L),tet(M), tet(45) (1)*<br>*aac(6′)-Ii ,ant(6)-Ia, eatAv, erm(B), pbp5, msr(C), tet(S), vat(E) (1)*<br>*aac(6′)-Ii, eatAv, efmA, erm(B),msr(C),tet(L),tet(M),tet(45) (1)*
Non-MDR	33 (53.2)	2 (3.2)		
MDR	29 (46.7)	60 (96.8)		
Resistance	62(100)	62(100)		

CIP, Ciprofloxacin; DAP, Daptomycin; ERY, Erythromycin; KAN, Kanamycin; LIN, Lincomycin; NIT, Nitrofurantoin; Q/D, Quinupristin/dalfopristin; TET, Tetracycline; TIG, Tigecycline; TYL, Tylosin tartrate
